# Carbon‐ion irradiation together with autophagy inhibition and immune checkpoint inhibitors protect against pancreatic cancer development in mouse model

**DOI:** 10.1002/jhbp.12148

**Published:** 2025-04-14

**Authors:** Makoto Sudo, Yaoyao Wang, Jingren Wang, Koubun Yasuda, Keiko Mitani, Shuhei Hayashi, Masaki Ohmuraya, Hiroko Tsutsui, Jiro Fujimoto

**Affiliations:** ^1^ Department of Genetics School of Medicine, Hyogo Medical University Hyogo Japan; ^2^ Department of Immunology School of Medicine, Hyogo Medical University Hyogo Japan; ^3^ Department of Gastroenterological Surgery School of Medicine, Hyogo Medical University Hyogo Japan; ^4^ Department of Microbiology School of Medicine, Hyogo Medical University Hyogo Japan; ^5^ International Tourism and Medical Studies School of Medicine, Hyogo Medical University Hyogo Japan; ^6^ Osaka Heavy Ion Therapy Center Osaka Japan

**Keywords:** autophagy, CD8^+^ T‐lymphocytes, heavy ion radiotherapy, pancreatic cancer, regulatory T cells

## Abstract

**Background:**

Pancreatic cancer remains fatal because of resistance to chemo‐, radio‐, and immunotherapies. Carbon‐ion radiotherapy (CIRT) has been beneficial for patients with pancreatic cancer. The purpose of this study was to identify the mechanism by which CIRT exerts its anticancer activity, particularly in combination with immunotherapy.

**Methods:**

We implanted murine pancreatic cancer cells treated with CIRT and autophagy inhibitor HCQ (CIRT+HCQ) into syngeneic mice, followed by the application of a regulatory T (Treg) cell blockade using immune‐checkpoint inhibitors. We compared CIRT+HCQ‐treated tumors with those implanted without any treatment. Further, we also implanted CIRT+HCQ‐treated pancreatic tumors into CD8^+^ T cell‐depleted mice. To characterize immunological alterations, we conducted immunohistology and flow cytometry of implanted tumors.

**Results:**

CIRT+HCQ‐treated tumors exhibited reduced growth, higher numbers of CD8^+^ T cells, and lower numbers of Treg cells compared with control tumors. CD8^+^ T cell depletion restored growth in CIRT+HCQ‐treated tumors. A Treg blockade resulted in greater tumor growth remission and elevated levels of intratumor CD8^+^ T cells in mice bearing CIRT+HCQ‐treated tumors but not in mice bearing control tumors.

**Conclusions:**

Treg cell‐targeted therapy exerted an anticancer effect in mice bearing CIRT+HCQ‐treated tumors but not in those bearing untreated pancreatic tumors by activating cancer‐specific CD8^+^ T cells.

## 1 INTRODUCTION

Pancreatic cancer is the sixth highest cause of deaths due to cancer worldwide.^1^, ^2^ The 5‐year survival rate for pancreatic cancer is still <10%.^3^, ^4^ As this cancer is often asymptomatic in the early stage, the first clinical visit of most patients results in a diagnosis of an advanced stage of pancreatic cancer. As in other types of cancer, resection remains the gold standard for pancreatic cancer treatment.^5^ Unlike other types of cancer, resection is followed by frequent recurrence and/or metastasis, even though the marginal regions of the resected pancreatic cancer mass are microscopically negative for cancer cells.^6^, ^7^ Patients with unresectable pancreatic cancer are treated with chemotherapy, radiotherapy, immunotherapy, or a combination of these, but these interventions have been shown to have mild efficacy, if any.^6^, ^8^, ^9^ Thus, pancreatic cancer therapy still faces great challenges.

Heavy‐ion radiotherapy has been reported to be effective in treating pancreatic cancer.^10^, ^11^, ^12^, ^13^ Since carbon ions, frequently used in heavy‐ion radiotherapy, have 12 times the mass of a proton, they generate high‐dose regions in the irradiated tumor. This attribute generates clustered DNA breaks, including double‐stranded DNA (dsDNA) breaks in cancer cells that damage them to an extent beyond their DNA repair ability. Carbon‐ion beams have unique physical properties including the spread‐out Bragg peak, which render the beam capable of reaching deeply embedded cancer cells and causing little damage to normal cells beneath the tumor.^14^ Based on these features, it is possible to irradiate deeply embedded pancreatic tumors with high‐dose carbon‐ion beams safely. It is intriguing to note that, like photon and proton irradiation, carbon‐ion beams have the potential to activate tumor‐targeted immunity.^15^ Carbon‐ion radiotherapy (CIRT) can evoke anticancer immunity, in particular tumoricidal CD8^+^ T cells.^15^ Thus, radiotherapy, including CIRT, exerts its anticancer action via direct, cancer cell‐targeted physical activity as well as indirect activation of anticancer immune responses.^15^ Simultaneously, CIRT potently induces autophagy in pancreatic tumors. Recent studies using murine pancreatic cancer models and clinical trials have unveiled a beneficial role of autophagy inhibitors in pancreatic cancer therapy.^16^, ^17^, ^18^

The tumor microenvironment (TME) is a platform that promotes tumor growth, metastasis, and resistance to therapy. Pancreatic cancer has a unique TME.^19^ Unlike many types of solid cancers, pancreatic cancer cells are nominally scattered in the dense and robust stroma consisting of fibroblasts, extracellular matrix proteinases, the vasculature, and various leukocyte types comprising robust pro‐cancerous immune cells, such as regulatory T cells (Treg), but few CD8^+^ T cells. These features may cause pancreatic cancer to become resistant to therapy.^16^ For example, a poor vasculature may hinder anticancer medicine from reaching cancer cells in sufficient quantity. The small numbers of intratumor CD8^+^ T cells may be insufficient for killing cancer cells even after depletion of pro‐cancerous immune cells by immunotherapy, such as immune checkpoint inhibitors (ICIs). Indeed, pancreatic cancer is reportedly resistant to monotherapeutic immunotherapy.^6^, ^8^ Here, we investigated whether CIRT‐treated pancreatic cancer cells resulted in poor tumor growth via activating anti‐pancreatic cancer immunity, and if so, how concurrent immunotherapy protected further against pancreatic cancer development.

## 2 METHODS

### 2.1 Cell culture and reagents

Pan02 is a well‐established mouse pancreatic ductal adenocarcinoma cell line derived from male C57BL/6 mice treated with 3‐methylcholanthrene^20^ and was purchased from the Developmental Therapeutics Program, Division of Cancer Treatment and Diagnosis, National Cancer Institute (Frederick, MD, USA). Pan02 cells were cultured in Dulbecco's modified Eagle's medium (Gibco Thermo Fisher Scientific‐JP, Tokyo, Japan) with 10% fetal bovine serum at 37°C and 5% CO_2_. Hydroxychloroquine (HCQ) was purchased from Cayman Chemical (Ann Arbor, MI, USA). For mouse CD8^+^ T cell depletion experiments, InVivoMab anti‐mouse CD8α and InVivoMab rat IgG2a isotype‐matched control were purchased from Bio X Cell (Lebanon, NH, USA). For Treg functional suppression experiments, InVivoMab anti‐mouse cytotoxic T‐lymphocyte‐associated protein (CTLA)‐4 (CD152) and control, InVivoMab polyclonal Syrian hamster IgG, were purchased from Bio X Cell. Antibodies against interleukin (IL)‐2Rα/CD25 (CST39475) and forkhead box P3 (FoxP3; Cat. # CST12653) were purchased from Cell Signaling Technology (MA, USA). Antibodies against CD4 (Cat. # ab183685) and CD8α (Cat. # ab217344) were purchased from Abcam (Cambridge, UK). For immunohistochemistry (IHC), EnVision+/HRP Rabbit (Cat. # K400311‐2) was purchased from Dako (Denmark). For multiple fluorescence staining, HRP anti‐rabbit IgG Donkey (711‐035‐152) secondary antibody was purchased from Jackson ImmunoResearch (West Grove, PA, USA). Opal 520, 570, and 690 fluorophores were purchased from Akoya Biosciences (Marlborough, CA, USA).

### 2.2 In vitro carbon‐ion irradiation

For in vitro carbon‐ion irradiation, cells cultured in T25 or T225 flasks (IWAKI AGC TECHNO GLASS, Shizuoka, Japan) were irradiated with 100 MeV horizontal carbon‐ion beams at the Osaka Heavy Ion Therapy Center (Osaka, Japan). The cell culture surface was placed at the Bragg Peak where the linear energy transfer was approximately 80 KeV/μm. The cells receiving suboptimal doses of CIRT (2 Gy) were incubated with HCQ, which resulted in moderate colony number reduction on day 14.^16^ In some experiments, the cells were pretreated with 10 μM HCQ prior to CIRT.

### 2.3 Mouse experiments

Previously we performed isograft in both male and female mice and found no differences in tumor growth formation between male and female mice. In this study, we chose to study female mice. Female C57BL/6J mice (6–8 weeks old) were purchased from Japan SLC, Inc. (Shizuoka, Japan). All animal experiments were performed under specific pathogen‐free conditions according to the guidelines of the Institutional Animal Care Committee of the Hyogo Medical University (Protocol Number: 20‐060).

Pan02 cells were cultured in four groups with four different protocols: untreated control, autophagy inhibitor (HCQ) alone, CIRT alone, and CIRT together with HCQ. After 24 h of incubation, the cells were harvested and injected with 1.0 or 2.0 × 106^6^ cells/shot into both flanks of the mice. Three weeks after inoculation (2.0 × 10^6^ cells/shot) or 6 weeks after inoculation (1.0 × 10^6^ cells/shot), the mice were killed, and their tumors were carefully dissected and weighed.^16^ Five mice were used for each experimental group.

Next, tumor‐infiltrating leukocyte analysis was carried out. Isograft tumors were dissociated into single‐cell suspensions using the BD Horizon Dri Tumor & Tissue Dissociation Reagent (BD Biosciences, San Diego, CA, USA), according to the instructions of the manufacturer. The cell suspension was stained for cell surface markers to identify the immune cells. Purified anti‐mouse CD16/32, PE anti‐mouse CD3ε, FITC anti‐mouse CD4, APC anti‐mouse CD8α, PE/Cyanine7 anti‐mouse CD25, APC/Cyanine7 anti‐mouse CD45, and Brilliant Violet 421 anti‐mouse FOXP3 were purchased from BioLegend (San Diego, CA, USA). Anti‐Mo NK1.1 PerCP‐Cyanine 5.5 was purchased from Invitrogen (Waltham, MA, USA). The samples were analyzed using the BD LSRFortessa flow cytometer with BD FACSDiva software.

CD8^+^ T cell depletion experiments were carried out using InVivoMab anti‐mouse CD8α and InVivoMab rat IgG2a isotype control. One day before the isograft, each mAb was intraperitoneally injected into mice at 250 μg/mouse. All injections were administered twice weekly for 3 weeks. Four mice were treated for each experimental group.

Treg functional suppression experiments were performed using InVivoMab anti‐mouse CTLA‐4 (CD152) mAb and InVivoMab polyclonal Syrian hamster IgG control Abs. Two days before the isograft, each antibody was intraperitoneally injected into mice at 150 μg/mouse. Immediately before and 2 days after the isograft, each mAb was intraperitoneally injected into mice at 150 μg/mouse. In total, three injections were administered. Four mice were used for each experimental group.

### 2.4 IHC

IHC was performed using pancreatic cancer specimens, as previously described.^16^ Briefly, the tissue specimens were fixed with IHC Zinc Fixative (BD Pharmingen|Becton, Dickinson and Company, Flanklin Lakes, NJ, USA) and embedded with paraffin wax before sectioning. The sections were preincubated with a serum‐free protein block (Agilent Technologies, Santa Clara, CA, USA) for 30 min at room temperature, and primary antibodies were added to each slide. Secondary Ab staining was conducted using Histofine Simple Stain Mouse MAX‐PO (Nichirei Biosciences Inc., Tokyo, Japan.), according to the instructions of the manufacturer. Finally, the stained slides were observed using the Eclipse Ni‐U microscope (Nikon, Tokyo, Japan).

### 2.5 Immunofluorescence analysis

Immunofluorescence analysis was performed as previously described.^21^ Briefly, tissue samples were fixed in IHC Zinc Fixative and embedded in paraffin wax. For multiple fluorescence staining, cell surface markers were detected by incubating the tumor slice with primary Ab (process 1). A secondary Ab (HRP anti‐rabbit IgG Donkey) reaction was carried out (process 2). Each fluorophore (Opal 570, 520, or 690) was conjugated (process 3). Processes 1, 2, and 3 were repeated. Fluorescence was visualized under a confocal microscope (Zeiss LSM780) and detected as follows: IL‐2Rα/CD25 (red), CD4 (green), and FoxP3 (cyan).

### 2.6 Statistical analyses

Multiple groups were compared using Tukey's test via a free online software (www.gen‐info.osaka‐u.ac.jp/MEPHAS/tukey.html), whereas two groups were compared using the Student's *t*‐test. Statistical significance was set at *p* < .05.

## 3 RESULTS

### 3.1 CIRT‐treated pancreatic cancer isograft shows impaired growth via accumulation of intratumor CD8 ^+^ T cells

**FIGURE 1:**
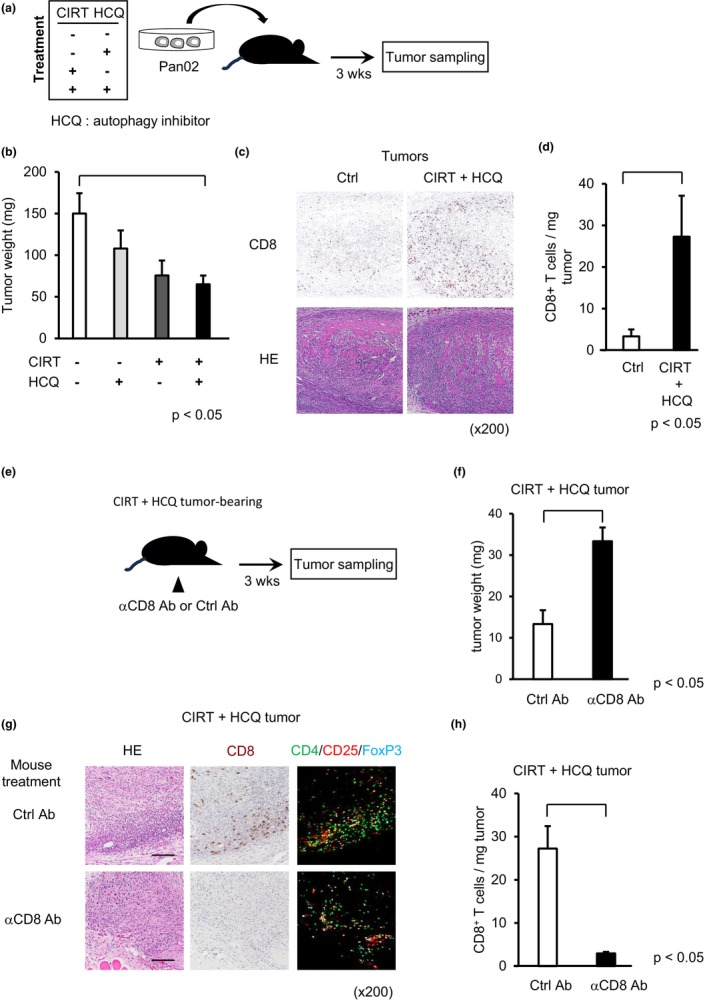
Requirement of CD8^+^ T cells for tumor suppression in CIRT+HCQ isografts. (a) We implanted mice with isografts of Pan02 cells treated with four types of combination treatments of CIRT and HCQ (*n* = 5) and sampled each tumor 3 weeks after implantation. (b) We weighed tumors derived from a control isograft of untreated Pan02 cells, of Pan02 cells treated with HCQ but not CIRT, of Pan02 cells treated with CIRT but not HCQ, or of Pan02 cells treated with both CIRT and HCQ. (c) We performed immunohistochemistry for CD8 in tumors of control isografts and tumors of isografts of Pan02 cells receiving both CIRT and HCQ (CIRT+HCQ). (d) We stained each tumor slice with hematoxylin and eosin (HE) as well. We counted CD8^+^ T cell numbers and calculated CD8^+^ T cells/tumor weight (mg). (e) To deplete CD8^+^ T cells, we administered anti‐CD8 Ab or isotype‐matched control Ab in mice bearing CIRT+HCQ isografts (*n* = 4) and sampled tumors 3 weeks after implantation. (f) We weighed tumors in control Ab‐ or anti‐CD8 Ab‐treated mice bearing CIRT+HCQ tumors. (g) We performed immunochemistry for CD8 (CD8^+^ T cells) and immunofluorescence staining for CD4 (green), CD25 (red), and Foxp3 (Treg cells in cyan) of CIRT+HCQ tumors of mice treated with anti‐CD8 Ab or control Ab. (h) CD8^+^ T cell numbers/tumor weight were calculated. Data are represented as the mean ± standard error of mean (SEM) of six tumors in each group. (c and g) Representative photos.

We wanted to know whether CIRT treatment of pancreatic cancer cells enhanced anticancer immunity under an intact immune system. To address this, we utilized a murine pancreatic cancer isograft model: Mice were inoculated with a syngeneic murine pancreatic cancer cell line, Pan02, to enable tumor development. As clinical doses of CIRT (55.2 Gy in 12 fractions) kill almost all human and murine pancreatic cancer cells, we irradiated Pan02 cells with a suboptimal dose (2 Gy). Previously, we had observed that CIRT induced pro‐cancerous autophagy, a known cellular survival mechanism, in human pancreatic cells.^16^ To evaluate the authentic anticancer activity of CIRT in the present study, we incubated the CIRT‐treated murine pancreatic cancer cells with an autophagy inhibitor (HCQ). We treated Pan02 cells with or without CIRT, followed by incubating them with or without HCQ. These four groups of Pan02 cells were injected into both flanks of syngeneic mice and developing tumors were harvested at week 3 post‐injection (Figure 1a). Tumors derived from isografts of Pan02 cells treated with either CIRT or HCQ alone showed slightly reduced growth compared with that of untreated control cells (denoted as control isografts); however, this difference was not statistically significant (Figure 1b) (control vs HCQ *p* = 0.4553, control vs CIRT *p* = 0.0523). However, the weights of tumors derived from isografts of Pan02 cells receiving both CIRT and HCQ (denoted as CIRT+HCQ isografts) were significantly less than those derived from control isografts (*p* = 0.0229) (Figure 1b). We compared tumors derived from CIRT+HCQ isografts and control isografts. IHC revealed a few CD8^+^ T cells and abundant CD8^+^ T cells in control and CIRT+HCQ tumors, respectively (Figure 1c). This pattern was also observed for intratumor CD8^+^ T cell numbers per tumor weight (Figure 1d). To investigate whether the accumulated CD8^+^ T cells were responsible for the tumor growth retardation observed in CIRT+HCQ tumors, we depleted CD8^+^ T cells by administering an anti‐CD8 mAb in mice bearing CIRT+HCQ tumors (Figure 1e). Expectedly, treatment with the anti‐CD8 mAb restored tumor growth as compared to isotype‐matched control mAb treatment (Figure 1f), indicating a contribution of CD8^+^ T cells to impaired growth in CIRT+HCQ tumors. IHC results confirmed that anti‐CD8 mAb treatment depleted the CD8^+^ T cell population in CIRT+HCQ tumors (Figure 1g,h). Taking together, these results suggested that CIRT+HCQ isografts may permit recruiting dense cancer‐specific CD8^+^ T cells, eventually leading to a retardation of tumor growth.

### 3.2 CIRT induction of MHC‐1 in pancreatic cancer cells

**FIGURE 2:**
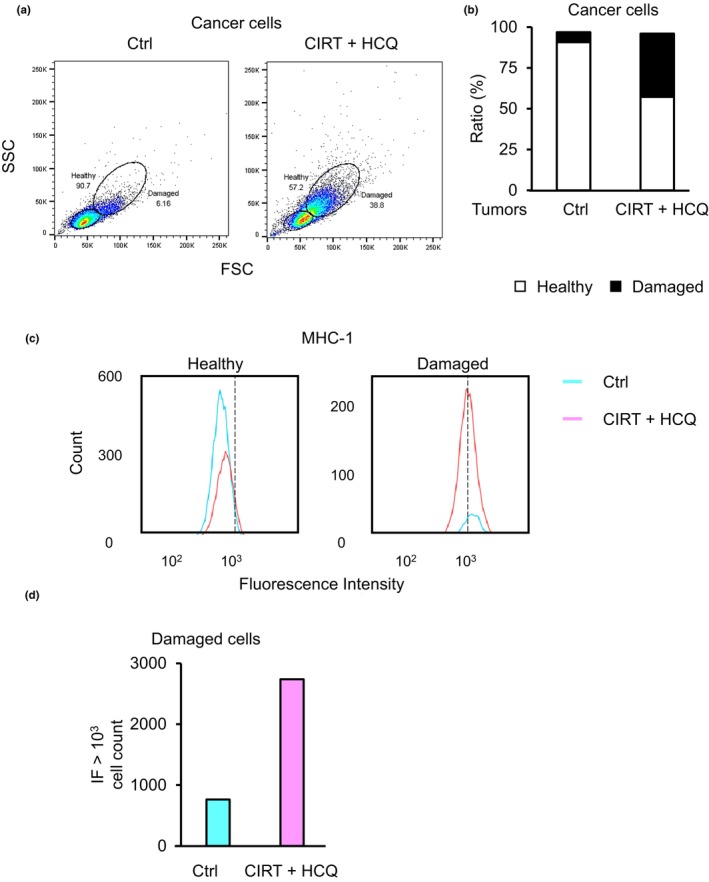
Treatment with CIRT+HCQ induces MHC‐I expression in pancreatic cancer cells. (a) We irradiated Pan02 cells with CIRT followed by incubation with HCQ. To measure MHC‐I expression, we carried out flow cytometry for Pan02 cells treated with CIRT+HCQ or untreated control cells. We gated healthy and damaged cells derived from CIRT+HCQ treated or untreated cells. (b) We measured cell numbers in each fraction of CIRT+HCQ treated and untreated control cells and calculated the healthy: Damaged cell ratio. (c) We measured MHC‐I expression levels in each fraction of CIRT+HCQ cells (red lines) and control cells (blue lines). Dashed lines show fluorescence intensity in 10^3^ fluorescence intensity units. (d) We measured cells expressing MHC‐I at levels of 10^3^ fluorescence intensity units or greater in the damaged cell population fraction of control cells (blue gar) and CIRT+HCQ cells (red bar). Similar results were obtained in two separate experiments.

It is well established that upon recognition of cancer‐derived neoantigen peptide in association with the major histocompatibility complex class 1 (MHC‐I) on cancer cells, cancer‐specific CD8^+^ T cells are activated to kill cancer cells.^22^ A massive amount of evidence supports the expression of low levels of MHC‐1 on the cell surface of various types of cancer cells.^23^ This allowed us to investigate whether MHC‐I expression was elevated in response to CIRT+HCQ Pan02 cells. As CIRT is capable of damaging cells,^14^, ^15^ we first determined the regions gating living cells and damaged cells using flow cytometry and then measured cell surface MHC‐I levels in each region. The region in which more than 90% of untreated control Pan02 cells were gated was estimated to be a healthy gate (Figure 2a, left panel). Less than 60% of CIRT+HCQ‐treated Pan02 cells were gated in the healthy region, indicating that the cells excluded from this region may be damaged. So, we denoted the region comprising cells excluded from the healthy region as the damaged gate (Figure 2a, right panel). Next, we measured MHC‐I expression levels of living and damaged CIRT+HCQ‐treated Pan02 cells in the healthy and damaged gates, respectively. Intriguingly, control Pan02 cells consisted of a major proportion of healthy cells and a minor proportion of damaged cells, whereas CIRT+HCQ‐treated cells were a mixture of healthy and damaged cells at an approximately 1:1 ratio (Figure 2a,b). Cells gated in the healthy region of control and CIRT+HCQ groups expressed MHC‐I at comparable fluorescence intensities but below 10^3^ fluorescence intensity units (Figure 2c). Many CIRT+HCQ‐treated cells expressed approximately 10^3^ fluorescence intensity units or more (Figure 2c). Considering the difference in the proportions of damaged cells between control and experimental groups (Figure 2b), we counted cells expressing MHC‐I at 10^3^ or more fluorescence intensity units (Figure 2d). CIRT+HCQ treatment especially induced MHC‐I expression in damaged cell populations. These results suggest that CIRT+HCQ treatment induces MHC‐I expression selectively in damaged cells, which presumably contributed to the activation of cancer‐specific CD8^+^ T cells. Thus far, our findings indicated that upon treatment with CIRT+HCQ, cancer cells expressed higher levels of MHC‐I, which in turn may have activated and expanded CD8^+^ T cells, eventually leading to the dampening of tumor growth.

### 3.3 Poor Treg cell accumulation in CIRT+HCQ tumors

**FIGURE 3:**
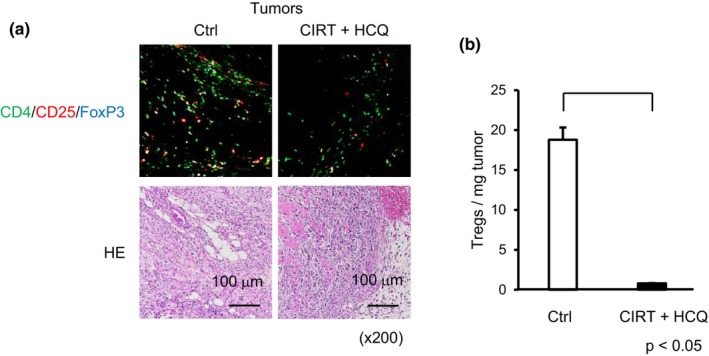
Reduction of intratumor Treg cell numbers in tumors derived from CIRT+HCQ isografts. (a) We carried out immunofluorescence staining for CD4^+^ CD25^+^ Foxp3^+^ Treg cells derived from control and CIRT+HCQ tumors. Representative photos of tumors are shown. (b) We calculated Treg cell numbers per tumor weight for control and CIRT+HCQ tumors. Data are presented as the mean ± standard error of mean (SEM) of six tumors in each group.

Pancreatic cancer is characterized by a unique TME composed of pro‐cancerous leukocytes, including Treg cells.^19^, ^24^ We investigated whether only cancer cells were capable of accumulating Treg cells. We performed immunofluorescence analysis in control tumors and found a dense accumulation of CD4^+^ CD25^+^ Foxp3^+^ Treg cells (Figures 1g and 3a,b; Figure S1), indicating that Pan02 cells harbor Treg‐accumulating capacity. In contrast, a few CD4^+^ CD25^+^ Foxp3^+^ Treg cells were observed to have infiltrated into CIRT+HCQ tumors (Figures 1g and 3a,b). Treg cells are capable of inhibiting CD8^+^ T cell function in terms of their expansion and cytotoxicity.^25^ Plausibly, poor Treg accumulation in CIRT+HCQ tumors may contribute to the activation and expansion of CD8^+^ T cells. Together, these data suggested that the impaired growth of CIRT+HCQ tumors may be due to both the induction of MHC‐I expression on cancer cells and an impaired infiltration of Treg cells.

### 3.4 Treg blockade enhances remission in CIRT+HCQ tumors but not in control tumors

**FIGURE 4:**
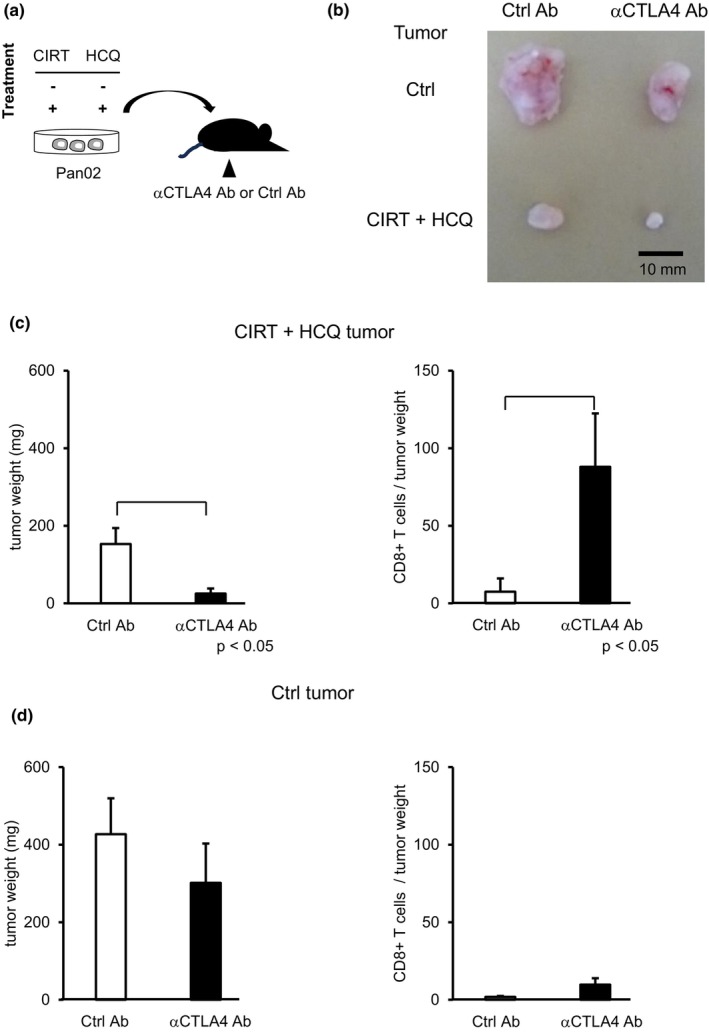
CTLA4‐checkpoint therapy further dampens tumor growth of CIRT+HCQ isografts but not of control isografts. (a) Mice implanted with control or CIRT+HCQ isografts were treated with anti‐ CTLA4 Ab or control Ab (*n* = 4) and tumors were sampled 3 weeks after implantation. (b) Representative photos of tumors are shown. We weighed tumors (left panel) and calculated intratumor CD8^+^ T cell numbers per tumor weight in mice implanted with CIRT+HCQ isografts (c) or those implanted with control isografts (d). Data are presented as the mean ± standard error of mean (SEM) of six tumors in each group.

CIRT+HCQ tumors contained abundant amounts of CD8^+^ T cells and small numbers of Treg cells, prompting us to investigate whether a blockade of these Treg cells may further protect against tumor growth by inducing activation and expansion of tumor‐infiltrating CD8^+^ T cells. To address this, we administered anti‐CTLA4 mAb or control Abs in mice bearing CIRT+HCQ isografts (Figure 4a). The administration of ICIs reduced tumor weights and increased CD8^+^ T cell numbers in CIRT+HCQ tumors compared with control Ab treatment (Figure 4b,c). In sharp contrast, treatment with anti‐CTLA4 mAb did not influence control tumors in terms of tumor growth and CD8^+^ T cell accumulation (Figure 4b,d). These results clearly indicated that CTLA4‐checkpoint therapy exerted its anticancer action selectively in CIRT+HCQ isograft‐bearing mice but not in control isograft‐bearing mice, presumably by activating and expanding cancer‐specific CD8^+^ T cells.

## 4 DISCUSSION

In this study, we found that CIRT+HCQ tumors were impaired in growth compared with control tumors and that their intratumor CD8^+^ T and Treg cell numbers were increased and decreased, respectively (Figure S2a,b). Importantly, CIRT enhanced the extracellular expression levels of MHC‐I on damaged cancer cells, which may have subsequently contributed to the activation and expansion of intratumor CD8^+^ T cells. CD8^+^ T cell depletion restored the growth of CIRT+HCQ tumors, indicating the importance of CD8^+^ T cells for impaired tumor growth (Figure S2a,b). Further, the application of a Treg cell blockade promoted a retardation in tumor growth and an accumulation of highly activated intratumor CD8^+^ T cells (Figure S2c).

DNA repair process produces an increase in MHC‐I expression in tumor cells.^26^ Photon or proton radiotherapy has the potential to activate DNA damage response signaling as well.^27^ Intriguingly, carbon‐ion beams have higher linear energy transfer (LET) than photon or proton beams.^27^ High LET radiotherapy induces robust DNA damage response signaling in tumors.^27^ This may imply that CIRT renders cancer cells expressing higher levels of MHC‐I than the other radiotherapies. Consistent with this, we found that CIRT induction of MHC‐I in pancreatic cancer cells, whereas photon and proton radiotherapy reportedly do not enhance MHC‐I levels in pancreatic cancer cells.^28^ However, it was recently reported that photon radiotherapy, proton radiotherapy, and CIRT cause comparable induction of MHC‐I in esophageal cancer cells.^29^ Thus, from the reports so far, we cannot simply conclude that CIRT is superior in MHC‐I induction in cancer cells to photon and/or proton therapy.

Our present study revealed that tumors of mice receiving CIRT+HCQ‐treated pancreatic cancer cells were sensitive to anti‐CTLA4 mAb, whereas those receiving untreated control cells were resistant to it, indicating that CIRT might render cancer cells sensitive to Treg blockade. Notably, after photon radiation, mice bearing pancreatic cancer cells become sensitive to Treg blockade as well.^30^ This clearly indicated that photon radiotherapy also has the potential to sensitize tumors to immunotherapy. However, we need further and careful studies to reveal whether or not, and if so, how radiation types determine the sensitivity of cancer to immunotherapy.

Recent studies clearly revealed a major mechanism underlying radiation activation of anticancer immunity. Radiotherapy of cancer cells generates cytoplasmic dsDNA breaks, which then activate the cyclic GMP–AMP synthase (cGAS)—stimulator of interferon gene (STING) pathway in the cells.^31^ Carbon‐ion beams generate dsDNA breaks as well, on a large scale.^32^ dsDNA‐derived activation of the cGAS‐STING pathway resulted in type‐I interferon (type I IFN) production.^31^ A large amount of evidence shows that type I IFN is capable of inducing MHC‐I expression.^33^ This may indicate that CIRT‐mediated type I IFN accounts for the enhanced MHC‐I levels on cancer cells, eventually resulting in activation and expansion of intratumor CD8+ T cells in CIRT+HCQ tumors. Indeed, in contrast to cells intact in type‐I IFN responsiveness, cancer cells null in it remain resistant to immunotherapy post radiotherapy.^34^ Type‐I IFN also directly activates CD8^+^ T cells to proliferate, differentiate into effector cells, and survive.^33^ Thus, CD8^+^ T cells in CIRT+HCQ tumors may contribute to impaired tumor growth, presumably via type‐1 IFN induced by CIRT.

CIRT+HCQ tumors harbored larger numbers of CD8^+^ T cells upon anti‐CTLA‐4 mAb treatment compared with those resulting from control mAb treatment (Figure 4). In contrast, control tumors continued to exhibit a few CD8^+^ T cells even after treatment with anti‐CTLA‐4 mAb. A few reasons may explain the resistance of control tumors to CTLA‐4‐checkpoint inhibitor therapy. First, intratumor CD8^+^ T cell numbers are small, and cancer cells express low levels of MHC‐I in control tumors. Under these conditions, tumor‐infiltrating CD8^+^ T cells seemed to lack the potential to respond to the neoantigen of the cancer, either in the presence or absence of a Treg cell blockade. Recently, it was reported that cancer irradiation induced clonal expansion of intratumor CD8^+^ T cells specific to the cancer.^30^, ^35^ Analogous to irradiation, CIRT is likely to expand anticancer CD8^+^ T cell clones, which may result in the generation of CD8^+^ T cell clonotypes that favorably and efficiently kill cancer cells. Thus, in combination with its induction of MHC‐I expression on cancer cells, CIRT‐derived generation of pinpoint‐specificity cancer‐killing CD8^+^ T cells may be reflected in the efficient retardation of CIRT+HCQ tumor growth. Second, control tumors lack the opportunity to develop diverse clonalities of CD8^+^ T cells.

CIRT+HCQ tumors display an impaired accumulation of Treg cells. We used Pan02 cells as pancreatic cancer cells in this study. Pan02 cells are a murine pancreatic cancer cell line generated from the pancreatic cancer of mice treated with a potent carcinogenic chemical and not by gene manipulations. To date, however, we do not know the gene mutations responsible for the intratumor accumulation of Treg cells in Pan02 cells. Additionally, little is known about how treatment with CIRT+HCQ insults tumor Treg infiltration. We need further studies to identify the mechanisms underlying the treatment with CIRT+HCQ that lead to a reduction in Treg recruitment.

We used a low dose CIRT. This prompted us to speculate that a low dose CIRT produces biological responses comparable with conventional radiotherapy. However, CIRT and conventional radiotherapy generate different types of DNA break damage in cancer cells.^27^ For example, conventional radiotherapy causes single‐strand DNA break damage majorly. In contrast, CIRT produces double‐strand DNA breaks robustly. Double‐strand DNA breaks are well‐known to be potent in activating the DNA repair response, while single‐strand DNA seems to be promptly repaired, eventually resulting in an infrequent DNA repair response. Based on the difference in physical features, tumor cell responses to CIRT even at a low dose we used in this study seem to be different from those to conventional radiotherapy.

It has been clearly shown that Kras mutations such as *Kras* ^*G12D*^ underly tumorigenesis of pancreatic cells and generation of their unique TME.^19^ In fact, pancreatic epithelial cell‐specific *Kras* ^*G12D*^ knock in mice spontaneously develop pancreatic cancer accompanied by a specific TME, whose characteristics resemble those of human pancreatic cancer.^36^ Various types of genetically engineered mice based on the *Kras* ^*G12D*^ knock in mice have revealed the precise process by which pancreatic cancer is generated and develops and that the *Kras* ^*G12D*^ mutation needs additional biological events to generate pancreatic cancer in adult mice. We found that the CIRT+HCQ treated tumor displayed a reduced number of Treg cells. To date, we have not been able to address the mechanism for this Treg cell reduction. If Pan02 cells develop pancreatic cancer depending on a Kras mutation, and if CIRT+HCQ‐treated tumors of *Kras* ^*G12D*^ isografts are impaired in Treg cell accumulation as well, we may be able to address how a Kras mutation is involved in this event precisely.

Recently, Yamamoto et al. reported that an autophagy inhibitor in combination with dual ICIs, anti‐CTLA4 Ab plus anti‐PD1 Ab, protected against pancreatic cancer growth in a murine isograft model.^23^ Their results appear to be different from ours. In this study, we found that treatment with CIRT+HCQ was necessarily required for pancreatic cancer cells to become susceptible to anti‐CTLA4 Ab. Several reasons may account for the difference between the results of Yamamoto et al. and our present findings. First, the study aims and experimental designs are different. Yamamoto et al. implanted murine pancreatic cancer cells into syngeneic mice and treated the mice daily with an autophagy inhibitor with or without treatment with ICIs after recognition of tumor development. In contrast, we injected mice with pancreatic cancer cells treated with CIRT together with HCQ as a temporal enhancer of the biological action of CIRT and sampled cancer mass on day 21 postimplantation, during which time we did not treat the mice with HCQ at all. Thus, frequent treatments with autophagy inhibitors may work as pancreatic cancer inhibitors^18^ in the previously published study, whereas our present data show that HCQ serves as an enhancer of the anticancer effects of CIRT. Second, the pancreatic cancer cells used in the two studies are different. Yamamoto et al. used a pancreatic cancer cell line generated from genetically engineered mice based on the pancreatic epithelial cell‐specific *Kras* ^*G12D*^ knock in mice. On the other hand, we utilized the murine Pan02 cell line as described above.

This study has some limitations. First, our present study is not directly translatable into clinical treatments. This is due to the possibility that patients with pancreatic cancer who will receive cancer therapies may have already developed immune responses to cancer antigens. In contrast, the host immune systems of mice receiving isografts are naïve to cancer antigens. Thus, the differences in immune systems may be critically important for designing a preclinical study. Second, we used the Pan02 pancreatic cancer cell line that was established from chemically induced pancreatic cancer in mice. It is unknown how widely Pan02 cells cover the genetic and biological features of human pancreatic cancers. We need further studies to identify the genetic and molecular characteristics of Pan02 cells to draw translational conclusions. Nevertheless, our present study enables us to conclude that treatment with CIRT renders pancreatic cancer cells susceptible to immunotherapy.

## FUNDING INFORMATION

This work was supported by the Japan Society for the Promotion of Scientific Research (JSPS) KAKENHI (Grant Number JP21H03011).

## CONFLICT OF INTEREST STATEMENT

The authors have no conflicts of interest to declare.

## Supporting information


Figure S1.

## Data Availability

The data that support the findings of this study are available on request from the corresponding author. The data are not publicly available due to privacy or ethical restrictions.
