# Supplementary material for: Carbon‐ion irradiation together with autophagy inhibition and immune checkpoint inhibitors protect against pancreatic cancer development in mouse model
Source: J Hepatobiliary Pancreat Sci. 2025 Apr 14;32(7):532–43. doi: 10.1002/jhbp.12148 (PMC12276459; doi:10.1002/jhbp.12148)
Supplement: Supplementary file 1 — Figure S1. [file JHBP-32-532-s001.pptx]

## Slide 1
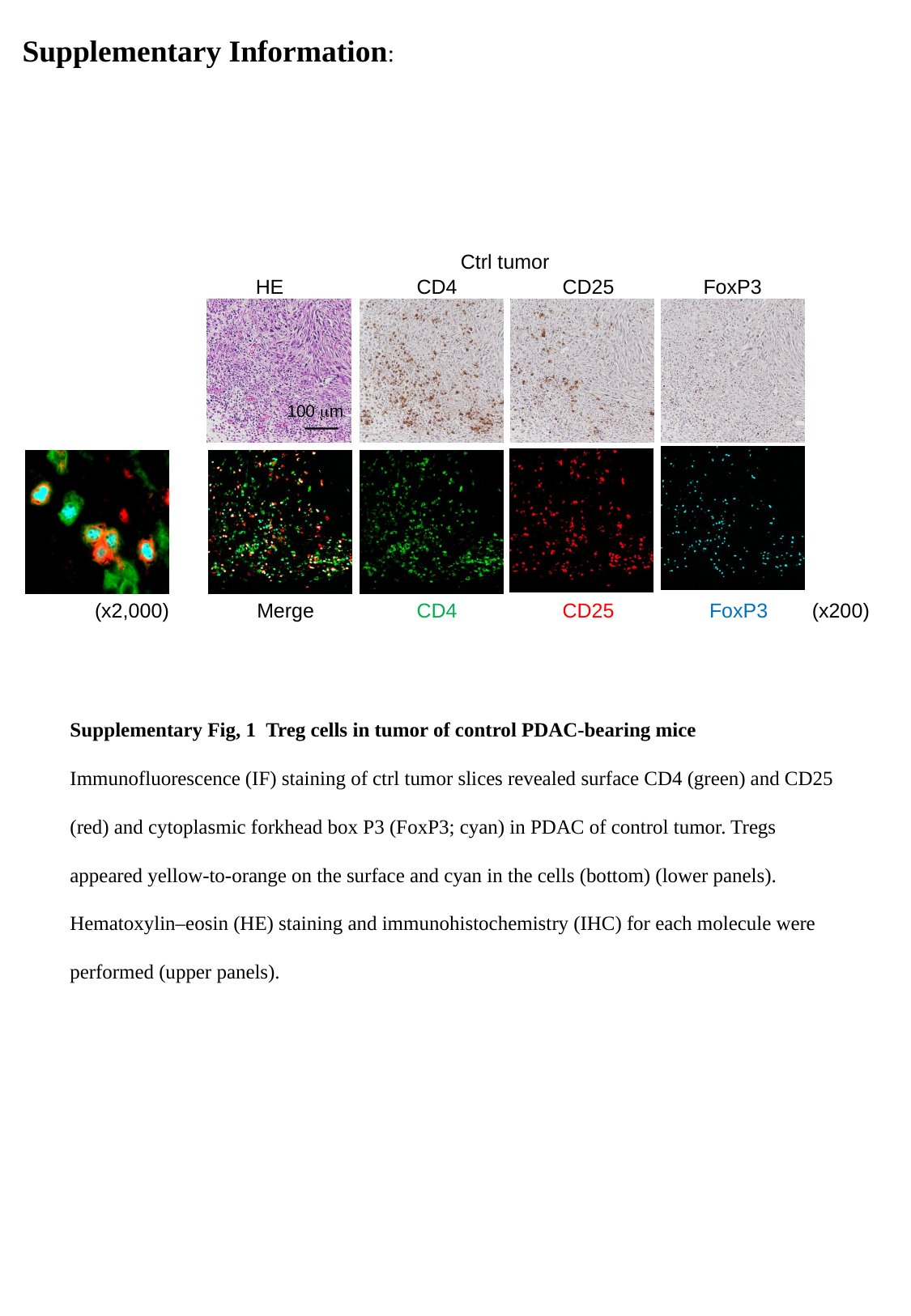

Supplementary Information:
Ctrl tumor
HE
CD4
CD25
FoxP3
100 mm
(x2,000)
Merge
CD4
CD25
FoxP3
(x200)
Supplementary Fig, 1 Treg cells in tumor of control PDAC-bearing mice
Immunofluorescence (IF) staining of ctrl tumor slices revealed surface CD4 (green) and CD25 (red) and cytoplasmic forkhead box P3 (FoxP3; cyan) in PDAC of control tumor. Tregs appeared yellow-to-orange on the surface and cyan in the cells (bottom) (lower panels). Hematoxylin–eosin (HE) staining and immunohistochemistry (IHC) for each molecule were performed (upper panels).

## Slide 2
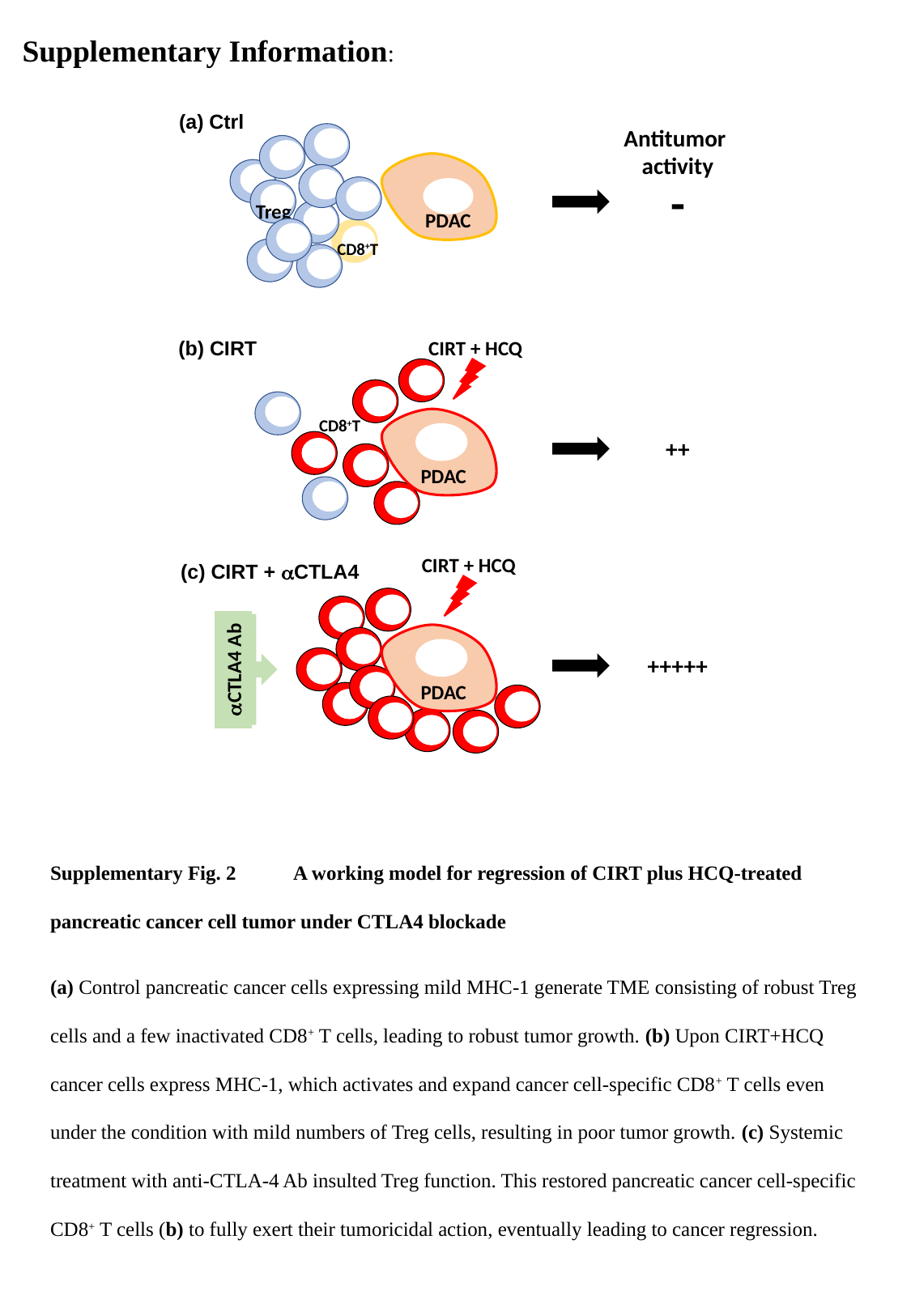

Supplementary Information:
(a) Ctrl
Antitumor
activity
Treg
PDAC
CD8+T
-
CIRT + HCQ
(b) CIRT
CD8+T
++
PDAC
CIRT + HCQ
aCTLA4 Ab
PDAC
(c) CIRT + aCTLA4
+++++
Supplementary Fig. 2	A working model for regression of CIRT plus HCQ-treated pancreatic cancer cell tumor under CTLA4 blockade
(a) Control pancreatic cancer cells expressing mild MHC-1 generate TME consisting of robust Treg cells and a few inactivated CD8+ T cells, leading to robust tumor growth. (b) Upon CIRT+HCQ cancer cells express MHC-1, which activates and expand cancer cell-specific CD8+ T cells even under the condition with mild numbers of Treg cells, resulting in poor tumor growth. (c) Systemic treatment with anti-CTLA-4 Ab insulted Treg function. This restored pancreatic cancer cell-specific CD8+ T cells (b) to fully exert their tumoricidal action, eventually leading to cancer regression.
